# Novel Proteins of the High-Affinity Nitrate Transporter Family NRT2, SaNRT2.1 and SaNRT2.5, from the Euhalophyte *Suaeda altissima*: Molecular Cloning and Expression Analysis

**DOI:** 10.3390/ijms25115648

**Published:** 2024-05-22

**Authors:** Dmitrii E. Khramov, Elena I. Rostovtseva, Dmitrii A. Matalin, Alena O. Konoshenkova, Olga I. Nedelyaeva, Vadim S. Volkov, Yurii V. Balnokin, Larissa G. Popova

**Affiliations:** K.A. Timiryazev Institute of Plant Physiology, Russian Academy of Sciences, Moscow 127276, Russia; khramov.de@yandex.ru (D.E.K.); ni-fir-titi@mail.ru (E.I.R.); matalin_net@mail.ru (D.A.M.); alenakonoshenkova@gmail.com (A.O.K.); olga.nedelyaeva@yandex.ru (O.I.N.); balnokin@mail.ru (Y.V.B.)

**Keywords:** halophyte, high-affinity nitrate transporter, nitrate, NRT2 proteins, *Suaeda altissima*

## Abstract

Two genes of nitrate transporters *SaNRT2.1* and *SaNRT2.5*, putative orthologs of high-affinity nitrate transporter genes *AtNRT2.1* and *AtNRT2.5* from *Arabidopsis thaliana*, were cloned from the euhalophyte *Suaeda altissima*. Phylogenetic bioinformatic analysis demonstrated that the proteins SaNRT2.1 and SaNRT2.5 exhibited higher levels of homology to the corresponding proteins from the plants of family Amaranthaceae; the similarity of amino acid sequences between proteins SaNRT2.1 and SaNRT2.5 was lower (54%). Both SaNRT2.1 and SaNRT2.5 are integral membrane proteins forming 12 transmembrane helices as predicted by topological modeling. An attempt to demonstrate nitrate transporting activity of *SaNRT2.1* or *SaNRT2.5* by heterologous expression of the genes in the yeast *Hansenula* (*Ogataea*) *polymorpha* mutant strain Δ*ynt1* lacking the only yeast nitrate transporter was not successful. The expression patterns of *SaNRT2.1* and *SaNRT2.5* were studied in *S. altissima* plants that were grown in hydroponics under either low (0.5 mM) or high (15 mM) nitrate and salinity from 0 to 750 mM NaCl. The growth of the plants was strongly inhibited by low nitrogen supply while stimulated by NaCl; it peaked at 250 mM NaCl for high nitrate and at 500 mM NaCl for low nitrate. Under low nitrate supply, nitrate contents in *S. altissima* roots, leaves and stems were reduced but increased in leaves and stems as salinity in the medium increased. Potassium contents remained stable under salinity treatment from 250 to 750 mM NaCl. Quantitative real-time PCR demonstrated that without salinity, *SaNRT2.1* was expressed in all organs, its expression was not influenced by nitrate supply, while *SaNRT2.5* was expressed exclusively in roots—its expression rose about 10-fold under low nitrate. Salinity increased expression of both *SaNRT2.1* and *SaNRT2.5* under low nitrate. *SaNRT2.1* peaked in roots at 500 mM NaCl with 15-fold increase; *SaNRT2.5* peaked in roots at 500 mM NaCl with 150-fold increase. It is suggested that SaNRT2.5 ensures effective nitrate uptake by roots and functions as an essential high-affinity nitrate transporter to support growth of adult *S. altissima* plants under nitrogen deficiency.

## 1. Introduction

Nitrogen is an important biogenic element. For terrestrial plants, the main source of nitrogen is nitrate, which is a dominating nitrogen form in aerated soils [1,2]. Plants absorb nitrate from the soil solution using specific transport mechanisms and molecular systems that function in their plasma membranes of root epidermal and cortical cells.

The availability of soil nitrate varies a lot due to its high turnover rate. It is explained by nitrate leaching, denitrification and different season-related edaphic factors [3]. Driven by the necessity to adapt to the wide range of changing nitrate concentrations, plants evolved transport systems with different affinities to nitrate [4,5]. According to the various concentrations of soil nitrate, the nitrate-transporting systems of plants could be divided to two groups, namely, low-affinity transport systems (LATS) and high-affinity transport systems (HATS). The LATS act at concentrations higher than 0.5 mM NO_3_^−^; the HATS act at low NO_3_^−^ concentrations (saturation in the range of 0.2 to 0.5 mM) [6].

Concentrations of nitrate present in soils are often in the micromolar range, which limits plant growth [5]. Low nitrate concentrations induce high-affinity transport systems (HATS) of plants; HATS allow for absorbing nitrate from the soil solution with concentrations below 0.5 mM [6,7]. Experiments with model salt-sensitive glycophyte *Arabidopsis thaliana* demonstrated that the important role in nitrogen supply under low nitrate concentration in the ambient medium is exhibited by the high-affinity nitrate transporters of the NRT2 family. Genes for seven members of the NRT2 family, *AtNRT2.1–AtNRT2.7*, were identified in the *Arabidopsis* genome [8]. The genes were found based on their similarity to *AtNRT2.1* [8,9]. The transporters of the NRT2 family of *Arabidopsis* are the best studied so far. It was demonstrated that the activity of high-affinity nitrate transporters AtNRT2.1, AtNRT2.2, AtNRT2.4 and AtNRT2.5 is localized in roots and linked to nitrate uptake [6,10,11,12]. Transporters AtNRT2.1 and AtNRT2.2 play an essential role under low nitrate concentrations [10,11]. Transporters AtNRT2.4 and AtNRT2.5 are also involved in high-affinity nitrate uptake but their activity is expressed under conditions of nitrate starvation only while AtNRT2.4 is important for nitrate uptake at very low external NO_3_^−^ concentrations [6,12]. Long-term nitrate starvation strongly induces the expression of *AtNRT2.5*; AtNRT2.5 brings the main contribution into high-affinity nitrate uptake under these conditions [6]. *AtNRT2.7* is specifically expressed in seeds; it is the only NRT2 transporter located in tonoplast for loading NO_3_^−^ into vacuoles [13].

Complete genome sequencing of plant species is rapidly progressing; 3517 genomes were sequenced from 1575 plant species from 2000 to 2024 [14]. This helped to discover many sequences encoding nitrate transporters of different families including NRT2. Five NRT2 genes have been identified in the rice genome [15]. Four ZmNRT2 genes have been identified in the maize genome (ZmNRT2.1, ZmNRT2.2, ZmNRT2.3 and ZmNRT2.5) [16]. Five nitrate transporter genes of the NRT2 family have been found in wheat (*Triticum urartu*) [17,18]. High-affinity nitrate transporters belonging to the NRT2 family were also found in a wide range of other higher plant species, including barley (*Hordeum vulgare*) [19], wild soybean (*Glycine soja*) [20], rapeseed (*Brassica napus*) [21], spinach (*Spinacea oleraecae*) [22] and *Poncirus trifoliata* [23].

Salinization reduces nitrate availability to plants. One of the main reasons is the competition between NO_3_^−^ and Cl^−^ for anion transporters [24,25,26]. For plants growing in soils with low (micromolar) nitrate concentrations, this could be especially crucial. The uptake of NO_3_^–^ and assimilation of nitrogen are suppressed in glycophytes under salinity [27].

It is hypothesized that halophytes, the plants naturally inhabiting saline soils, absorb nitrate under salinity more efficiently than glycophytes [28,29]. Anion-transporting proteins in the plasma membrane of halophyte root cells function when exposed to much more concentrated solutions of Na^+^ and Cl^−^ ions than the orthologous proteins of glycophytes, suggesting structural peculiarities for these proteins of halophytes and, correspondingly, their distinct physicochemical properties compared to the glycophytic orthologs. The characteristics ensure that the anion transport systems of halophytes bind nitrate and transport it via plasmalemma when nitrate concentration is orders of magnitude lower than the concentration of chloride. So, understanding the effects of salinity on nitrogen uptake in halophytes may be beneficial for creating salinity-tolerant agricultural plants. Nevertheless, available information about anion-transporting proteins of halophytes is scarce.

It is reasonable to assume that under the conditions of nitrate deficiency and at the same time of salinity with high (over 200 mM) NaCl in nutrient or soil solution, the key role in nitrate uptake and transport in plants is played by specific high-affinity nitrate transporters. Here, we describe the cloning of coding sequences for two high-affinity nitrate transporter genes, *SaNRT2.1* and *SaNRT2.5,* from the euhalophyte *Suaeda altissima* (Amaranthaceae, Suaedoideae). Many members of the genus *Suaeda* inhabit highly saline soils and are characterized by extreme salt tolerance, above 750 mM NaCl [30,31,32]. *S. altissima* Pall. is a herbaceous halophytic plant with very high salinity tolerance, sharing the same halophyte features as other representatives of the genus Suaeda. It is one of the most salt tolerant plants, able to complete its life cycle at 1 M NaCl concentrations [33]. Under natural conditions, *S. altissima* inhabits the shores of the largest salt lake in Europe, Elton.

Newly identified nitrate transporters from *S. altissima*, SaNRT2.1 and SaNRT2.5, are putative orthologs of high-affinity nitrate transporters AtNRT2.1 and AtNRT2.5 from *A. thaliana*. The relative abundance of *SaNRT2.1* and *SaNRT2.5* transcripts in *S. altissima* organs was measured for plants grown at various nitrate and chloride concentrations in nutrient solutions. The ability of SaNRT2.1 and SaNRT2.5 to transport nitrate was examined in a heterologous system, by functional complementation analysis in the mutant strain *Δynt1* of yeast *Hansenula (Ogatae) polymorpha*. *H. polymorpha* is a suitable model organism to study heterologous plant nitrate-transporting mechanisms since it is able to take up and metabolize nitrate as the only nitrogen source [34,35]. Gene *YNT1* (yeast nitrate transporter 1) encodes the only high-affinity nitrate transporter in *H. polymorpha* [36]. In the mutant strain *Δynt1*, used in this work, the gene *YNT1* is deleted.

## 2. Results

### 2.1. Growth Parameters and Anion Accumulation in S. altissima Organs

Quantitative analyses were completed to determine the effects of low nitrate (0.5 mM NO_3_^−^) and high nitrate (15 mM NO_3_^−^) conditions and NaCl treatments on the overall growth of *S. altissima* plants after 6 weeks in hydroponics.

**Figure 1:**
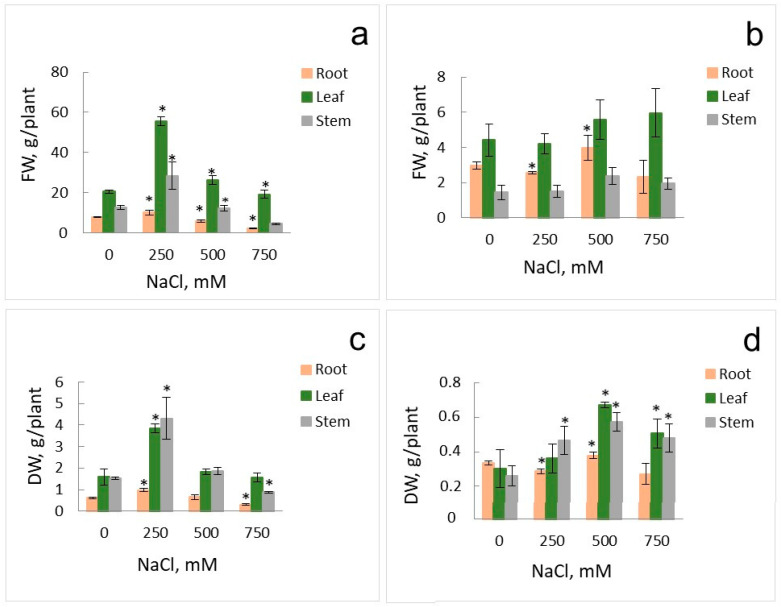
Fresh weights (FWs) (**a**,**b**) and dry weights (DWs) (**c**,**d**) of organs (roots, leaves, stems) of *S. altissima* plants grown at high nitrate (15 mM) (**a**,**c**) or low nitrate (0.5 mM) (**b**,**d**) and at various NaCl concentrations in the nutrient solution. A *p*-value < 0.05 was considered statistically significant. * *p* < 0.05. Standard deviations are given.

According to expectations, the growth of plants under low nitrate conditions was essentially reduced compared to the conditions when plants grew under high nitrate conditions (Figure 1). Addition of NaCl to the nutrient solution stimulated growth of the euhalophyte under both low nitrate (0.5 mM NO_3_^−^) and high nitrate (15 mM NO_3_^−^) conditions; the stimulation was strikingly significant when 250 mM NaCl was added to the ambient medium with 15 mM NO_3_^−^. Under low nitrate conditions, the growth of plants was maximal under 500 mM NaCl added (Figure 1).

**Figure 2:**
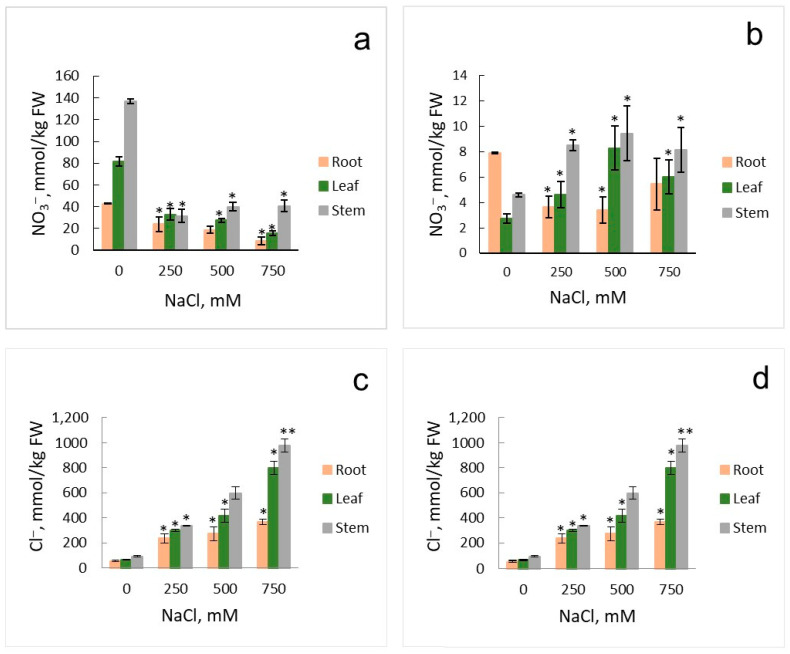
Nitrate (NO_3_^−^) (**a**,**b**) and chloride (Cl^−^) (**c**,**d**) contents in the organs (roots, leaves, stems) of *S. altissima* plants grown at high nitrate supply of 15 mM (**a**,**c**) or low nitrate supply of 0.5 mM (**b**,**d**) and at various NaCl concentrations in the nutrient solution. A *p*-value < 0.05 was considered statistically significant. * *p* < 0.05, ** *p* < 0.01. Standard deviations are given.

**Figure 3:**
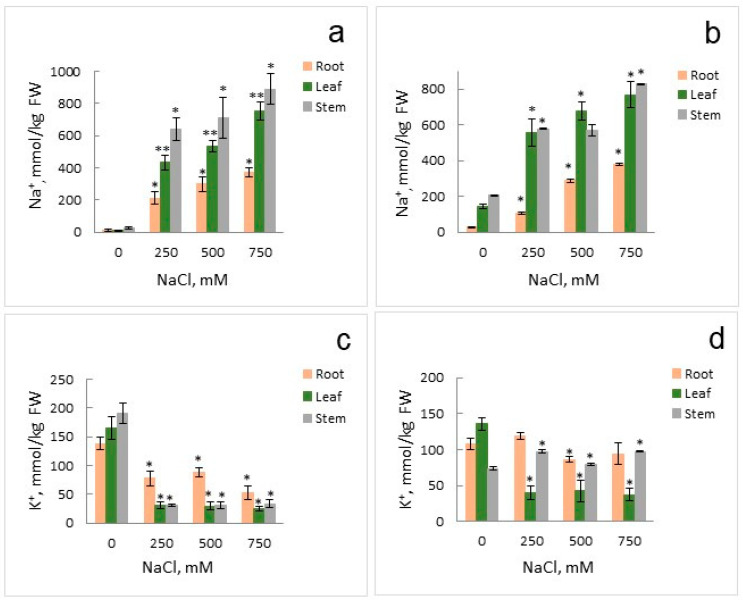
Sodium (Na^+^) (**a**,**b**) and potassium (K^+^) (**c**,**d**) contents in the organs (roots, leaves, stems) of *S. altissima* plants grown at high nitrate supply of 15 mM (**a**,**c**) or low nitrate supply of 0.5 mM (**b**,**d**) and at various NaCl concentrations in the nutrient solution. A *p*-value < 0.05 was considered statistically significant. * *p* < 0.05, ** *p* < 0.01. Standard deviations are given.

Measurements of nitrate contents in organs of *Suaeda* plants that grew under different concentrations of nitrate and chloride (NaCl) in the medium demonstrated that nitrate accumulated in high amounts in leaves and stems of plants growing in the medium with high nitrate and without NaCl (Figure 2a). Addition of 250 mM NaCl to the nutrient solution led to considerable drop in nitrate contents in organs of the euhalophyte. The further increase in NaCl in the nutrient solution resulted in the gradual decrease in nitrate in roots and leaves of *Suaeda* but nitrate contents remained about the same in stems of the plants. High nitrate contents in organs of euhalophyte grown in the nutrient solution without NaCl could be explained by the strategy of *S. altissima* to accumulate ions in vacuoles of cells in leaves and stems to maintain their low osmotic potential [33]. Sodium and chloride ions are accumulated in vacuoles of leaves and stems when NaCl is present in the nutrient solution while K^+^ and nitrate presumably substitute them in the absence of NaCl (Figure 2 and Figure 3).

The nitrate contents in the organs of *S. altissima* plants were low for low nitrate concentrations (0.5 mM) in the medium (Figure 2b), it corresponded to the reduced growth of the euhalophyte (Figure 1b,d). However, under the conditions of low nitrate in the nutrient solution, the increase in NaCl in the medium did not decrease but slightly increased total nitrate contents in the organs of the plants (Figure 2b), suggesting the functioning of specialized high-affinity nitrate transporting systems in roots of *S. altissima* that are able to bind and transport nitrate against the background of high external chloride concentrations.

### 2.2. Identification of the Full-Length Coding Sequences SaNRT2.1 and SaNRT2.5 for High-Affinity Nitrate Transporters and In Silico Analysis of the Protein Structures

Earlier, we identified the partial coding sequences (CDSs) of *SaNRT2.1* and *SaNRT2.5* (GenBank IDs: MK580128.1 and MK580129.1, accordingly), genes of high-affinity nitrate transporters from *S. altissima* [37]. Identification of the partial CDS of *S. altissima NRT2* genes was carried out assuming similarity of putative *S. altissima* genes with homologous genes from the halophytes *S. fruticosa* and *S. glauca*, which are closely related to *S. altissima*. Coding nucleotide sequences of *S. fruticosa* and *S. glauca* homologous genes were obtained by in silico analysis of the de novo assembled transcriptomes of these halophytes. The short-read RNA arrays for the transcriptome assembling were taken by us from the BioProject database, portal NCBI (Acc. No. #PRJNA279962, https://www.ncbi.nlm.nih.gov/bioproject/?term=PRJNA279962, accessed on 22 March 2024, and #PRJNA295637, https://www.ncbi.nlm.nih.gov/bioproject/?term=PRJNA295637, accessed on 22 March 2024).

**Figure 4:**
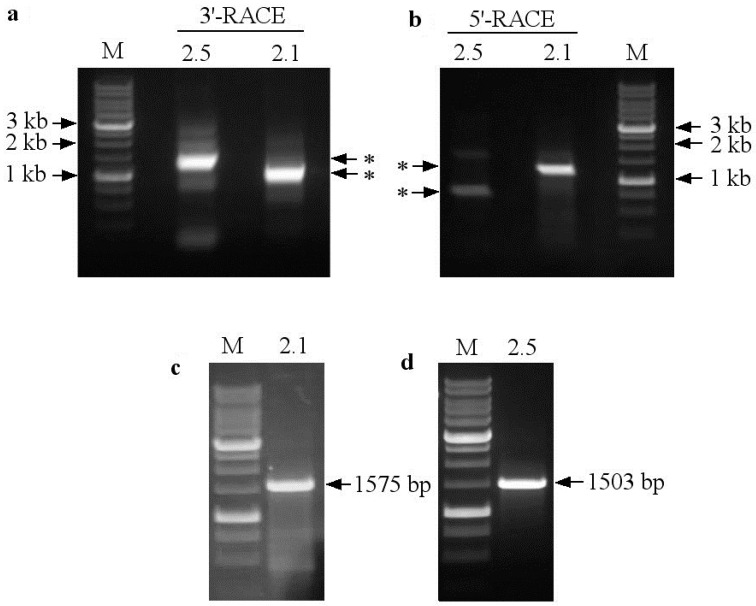
(**a**,**b**): Analysis of 3′-end and 5′-end fragments (indicated by arrows with asterisks) of *SaNRT2.1* and *SaNRT2.5* coding sequences synthesized on the total cDNA template from *S. altissima* roots using Step-Out RACE technology. (**c**,**d**): Analysis of the full-length *SaNRT2.1* (**c**) and *SaNRT2.5* (**d**) coding sequences that were used for further cloning in vector pCHLX. DNA fragments were separated by electrophoresis in 1% agarose gel. M—DNA molecular weight markers.

Here, based on the partial *SaNRT2.1* and *SaNRT2.5* CDSs, we obtained 3′- and 5′-end sequences of their cDNAs (Figure 4a,b). Then, using the experimental approach described in “Materials and Methods”, the complete cDNA sequences for the genes *SaNRT2.1* and *SaNRT2.5* were obtained from *S. altissima* (Figure 4c,d). Coding sequences *SaNRT2.1* (1575 bp) and *SaNRT2.5* (1503 bp) were cloned into yeast shuttle vector pCHLX, verified by sequencing and deposited in GenBank (*SaNRT2.1* ID: OR909030.1; *SaNRT2.5* ID: OR828748.1). *SaNRT2.1* encodes the protein of 524 amino acids with predicted molecular mass of 56.96 kDa, and *SaNRT2.5* encodes the protein of 500 amino acids with predicted molecular mass of 54.38 kDa.

**Figure 5:**
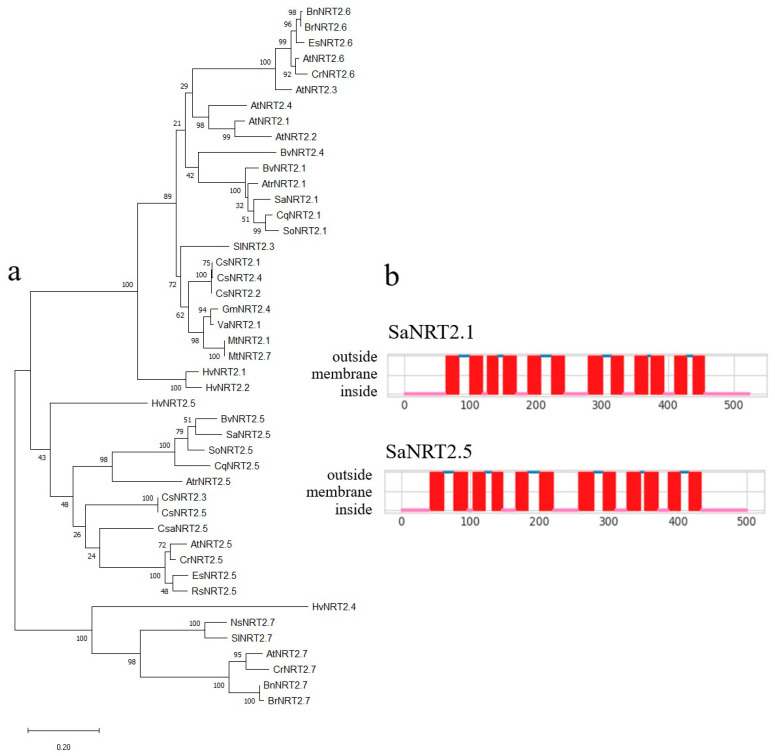
(**a**) An unrooted phylogenetic tree of the SaNRT2.1 and SaNRT2.5 transporters of *S. altissima* and other plant NRT2 homologs. The phylogenetic tree was built in MEGA 11 using the maximum likelihood method based on the Jones–Taylor–Thornton model. The number of bootstrap replicates was 1000; the values of bootstrap support are indicated near the nodes. The NRT2 protein sequences were extracted from the NCBI portal (https://www.ncbi.nlm.nih.gov/protein, accessed on 14 March 2024). Names of plant species and protein IDs are given in Table S2. (**b**) Membrane topology of proteins SaNRT2.1 and SaNRT2.5 predicted using DeepTMHMM software version 1.0.24. Both SaNRT2.1 and SaNRT2.5 form 12 transmembrane helices; N- and C-ends are located in cytoplasm.

In silico analysis carried out using the online resource InterPro (version 98.0, http://www.ebi.ac.uk/interpro/, accessed on 22 March 2024) as well as Protein BLAST at the NCBI portal (https://blast.ncbi.nlm.nih.gov/Blast.cgi, accessed on 22 March 2024) confirmed that the two identified transporters belong to the family of high-affinity nitrate transporters NRT2. In a phylogenetic tree based on amino acid sequences, SaNRT2.1 is located within a clade with NRT2.1 proteins from other Amaranthaceae—*Amaranthus tricolor*, *Beta vulgaris*, *Chenopodium quinoa*, *Spinacea oleracea* (Figure 5a). Accordingly, SaNRT2.5 lies in a clade with NRT2.5 proteins from the same plant species (Figure 5a).

The online service WoLF PSORT II predicted that both SaNRT2.1 and SaNRT2.5 resided in the plasma membrane. According to the topology model predicted using DeepTMHMM software (version 1.0.24), both SaNRT2.1 and SaNRT2.5 are integral membrane proteins forming 12 hydrophobic transmembrane helices (TMH); both N- and C-ends are in the cytoplasmic compartment (Figure 5b). Every six transmembrane helixes forming a group are connected by a central cytoplasmic loop, which is relatively short in *Suaeda* NRT2s. Such topology is generally typical for proteins of the MFS (major facilitator superfamily) [38]. MFS transporters are secondary carriers consisting of a single polypeptide capable of transporting small molecules of solutes in response to chemiosmotic ion gradients. They function as uniporters, symporters or antiporters. MFS proteins contain 12 transmembrane regions. Proteins of this superfamily are involved in the absorption of nitrate from the soil by plant roots.

**Figure 6:**
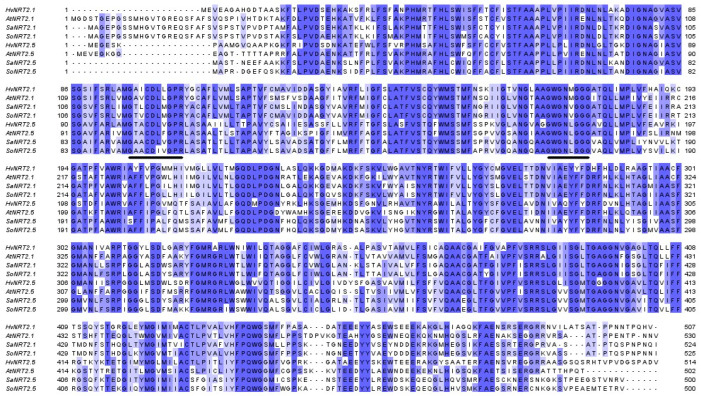
Multiple sequence alignment performed in Clustal Omega software (https://www.ebi.ac.uk/jdispatcher/msa/clustalo, accessed on 25 March 2024) for NRT2 proteins from *Arabidopsis thaliana* (AtNRT2.1: NP_172288.1, AtNRT2.5: NP_172754.1), *Hordeum vulgare* (HvNRT2.1: AAC49531.1, HvNRT2.5: KAE8819762.1), *Suaeda altissima* (SaNRT2.1: WPS65192.1, SaNRT2.5: WPH61290.1) and *Spinacia oleracea* (SoNRT2.1: XP_021865042.1, SoNRT2.5: XP_021845686.1). Protein GenBank IDs are indicated in the parenthesis. The key motifs (GxxxDxxGxR, GWGN(M/L)GGG) are marked by lines below the sequences. The intensity of the staining of amino acid residues corresponds to the degree of their identity (percentage identity).

The SaNRT2.1 and SaNRT2.5 sequences were aligned with NRT2 sequences from some other plants (Figure 6). The alignment reveals a number of conservative motifs in the polypeptide chains and among them the MFS-conserved motif G-xxx-D-xx-G-x-R and nitrate–nitrite transporter family motif G-W/L-G-N-M/L-G-G-G [39]. Table 1 shows the similarity of proteins SaNRT2.1 and SaNRT2.5 to each other and to proteins of the NRT2 family from *A. thaliana*. Similarity of SaNRT2.1 to SaNRT2.5 is not high: about 50%. Protein SaNRT2.1 demonstrates higher similarity to *Arabidopsis* NRT2 proteins with the exception of the AtNRT2.7 than to halophytic protein SaNRT2.5. The similarity of SaNRT2.5 to *Arabidopsis* NRT2 proteins is about the same as to SaNRT2.1. It is worth noting that AtNRT2.5 is also the most divergent protein of the whole AtNRT2 protein family where the genes are expressed in roots (Table 1).

### 2.3. Quantitative Analysis of SaNRT2.1 and SaNRT2.5 Transcripts in S. altissima Organs

**Figure 7:**
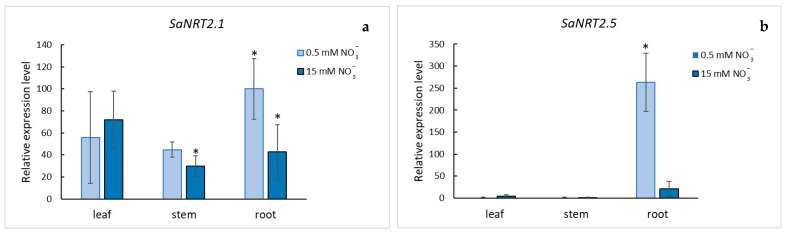
Relative abundance of *SaNRT2.1* (**a**) and *SaNRT2.5* (**b**) transcripts in the organs of *S. altissima* plants grown in the nutrient solution containing low (0.5 mM) or high (15 mM) NO_3_^−^ concentrations. There was no NaCl in the medium. The relative abundance of *SaNRT2.1* transcripts in the roots of the plants grown in the nutrient medium containing 0.5 mM NO_3_^−^ was taken as 100 percent. A *p*-value < 0.05 was considered to be statistically significant. * *p* < 0.05. Standard deviations are given.

**Figure 8:**
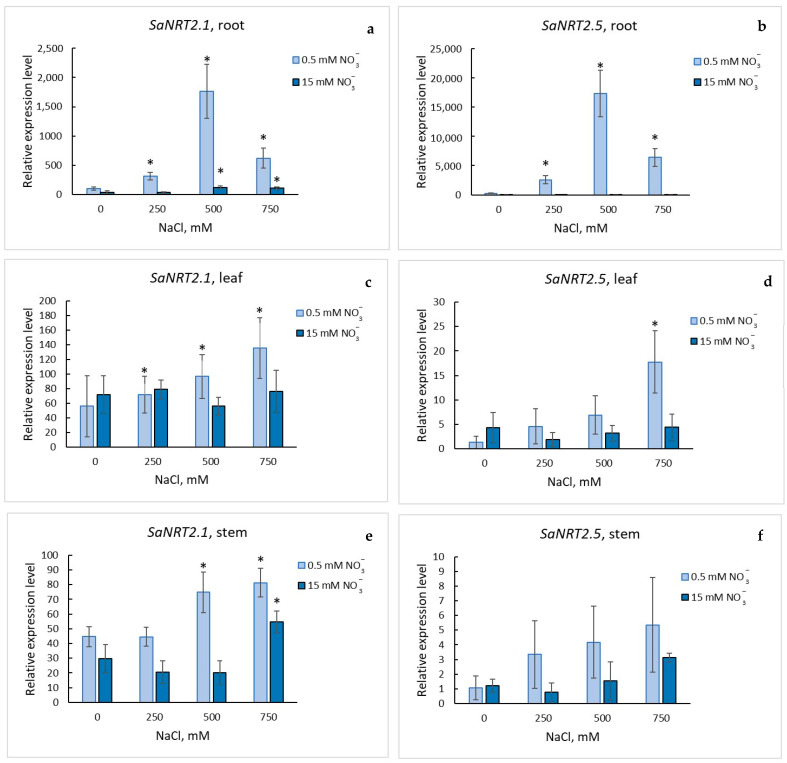
Relative abundance of *SaNRT2.1* (**a**,**c**,**e**) and *SaNRT2.5* (**b**,**d**,**f**) transcripts in the organs of *S. altissima* plants (roots (**a**,**b**), leaves (**c**,**d**), stems (**e**,**f**)) grown in the nutrient medium containing low (0.5 mM) or high (15 mM) NO_3_^−^ concentrations under increasing NaCl concentrations. The relative abundance of *SaNRT2.1* transcripts in the roots of the plants grown in the nutrient medium without NaCl and containing 0.5 mM NO_3_^−^ was taken as 100 percent. A *p*-value < 0.05 was considered to be statistically significant. * *p* < 0.05. Standard deviations are given.

We investigated expression of *SaNRT2.1* and *SaNRT2.5* in organs of *S. altissima* under different nitrate concentrations in the NS and different salinities. Observed patterns of *SaNRT2.1* and *SaNRT2.5* expressions were different (Figure 7 and Figure 8).

The levels of *SaNRT2.1* expression were comparable in all the studied organs; in roots, leaves and stems of the halophyte; independently of nitrate supply (low or high supply) when NaCl was absent in the nutrient solution (Figure 7a). An increase in NaCl concentrations in the nutrient solution changed the pattern. The expression of *SaNRT2.1* in the roots of plants under low nitrate essentially increased; it reached maximum at 500 mM NaCl (Figure 8a). The expression of *SaNRT2.1* changed insignificantly in leaves and stems under the conditions (Figure 8c,e). Under high nitrate concentrations in the nutrient solution, *SaNRT2.1* expression in roots increased but remained at a low level compared to the expression observed at low nitrate concentration; the expression of *SaNRT2.1* in leaves and stems was nearly not influenced by the treatment (Figure 8a,c,e).

*SaNRT2.5* was expressed mainly in roots of *S. altissima*; the expression of the gene in the other organs of *S. altissima* was negligibly low (Figure 7b). The level of *SaNRT2.5* expression in the roots was essentially influenced by concentrations of nitrate and chloride in the nutrient solution. In the absence of NaCl in the nutrient solution, the expression of *SaNRT2.5* was significantly reduced at high nitrate concentrations (Figure 7b) and was nearly completely suppressed with rising NaCl concentrations (Figure 8b). On the contrary, the expression of *SaNRT2.5* in the roots of plants that were grown under nitrate deficiency dramatically increased with the rise in NaCl; the maximum expression was achieved at 500 mM NaCl, similar to the expression of *SaNRT2.1* (Figure 8b). Expression of *SaNRT2.5* in other *S. altissima* organs remained negligibly low as the concentration of NaCl in the medium increased (Figure 8d,f)

It should be noted that the level of *SaNRT2.5* expression was 10 times higher than the level of *SaNRT2.1* expression when comparing expression of *SaNRT2.1* and *SaNRT2.5* genes in roots of *S. altissima* plants that were grown in the medium with NaCl under nitrate deficiency (Figure 8a,b).

### 2.4. Experiments on Functional Complementation of Yeast Mutant Δynt1 by SaNRT2.1 and SaNRT2.5 Expression in H. polymorpha Cells

**Figure 9:**
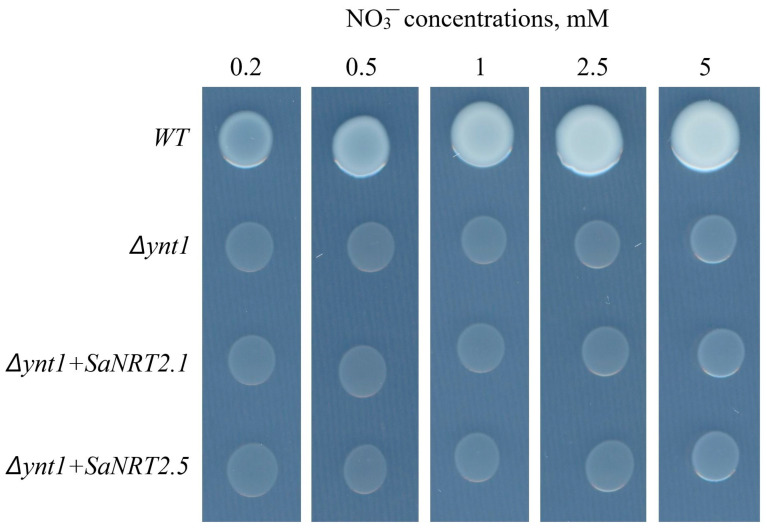
Complementation assay of the *H. polymorpha* mutant strain *Δynt1* transformed by pCHLXSaNRT2.1 or pCHLXSaNRT2.5 constructs during growth on minimal agarized SD medium supplied with different concentrations of nitrate (from 0.2 to 5 mM). Wild-type yeast strain DL-1 and the *Δynt1* mutant transformed with the empty vector pCHLX were taken as controls. Approximately 10^5^ cells of each strain were plated on Petri dishes and incubated at 37 °C for 3 days.

In order to demonstrate the nitrate transporting function of the SaNRT2.1 and SaNRT2.5 proteins, knockout mutant strain *Δynt1* of the methylotrophic yeast *H. polymorpha* was transformed with yeast integrative vector constructs pCHLXSaNRT2.1 or pCHLXSaNRT2.5, carrying the coding sequences *SaNRT2.1* or *SaNRT2.5*, respectively. Unlike the yeast wild-type (WT) strain, the growth of the mutant *Δynt1* strain lacking the only high-affinity nitrate transporter YNT1 was suppressed on a minimal SD medium containing NO_3_^−^ at concentrations ranging from 0.2 to 5 mM (Figure 9). Unfortunately, there was no noticeable recovery in the growth of the mutant *Δynt1* strain transformed by the vectors with coding sequences of heterologous nitrate carriers SaNRT2.1 or SaNRT2.5 (Figure 9). The reasons for this will be discussed in the Discussion Section.

## 3. Discussion

Nitrogen is an essential and one of the most important mineral nutrients for plants. The growth of *S. altissima* under the controlled experimental conditions of hydroponics demonstrated that low concentrations of nitrate in the nutrient solution (0.5 mM) expectedly reduced plant growth, by about five times in fresh and dry weights of shoots and stems, but less for roots, compared to plants growing at high nitrate concentrations (15 mM) (Figure 1). Concentrations of nitrate in *S. altissima* plant organs were also markedly reduced under low nitrate, by 5 times for roots and by over 20 times for leaves and stems (Figure 2a,b). However, contrary to glycophytes, the growth of euhalophyte *S. altissima* was significantly stimulated by increasing NaCl concentrations in the nutrient solution, peaking at 250 mM NaCl for high nitrate supply and even at 500 mM NaCl for low nitrate (Figure 1).

Experiments with species of the *Suaeda* genus, *S. physophora* and *S. salsa*, demonstrated that NaCl application significantly increased leaf NO_3_^−^ concentrations under N-sufficient conditions thus indicating that NaCl may have a promoting effect on nitrate uptake in some halophytes [29,40]. Different results were obtained in our experiments. The concentration of nitrate in *S. altissima* organs dropped 2–4 times under salinity for high nitrate supply though still remained higher than under low nitrate treatment and did not essentially decrease with increasing salinity up to 750 mM NaCl (Figure 2a). This is the typical effect for most plants under salinity (e.g., [27] for rice and tomato and references therein). However, although increasing salinity treatment did not reduce nitrate concentrations (apart from a slight initial decrease in the roots) in *S. altissima* organs under low nitrate, an increase was observed in the leaves and stems (Figure 2b), similar to findings with halophytes *S. physophora* and *S. salsa* [29,40]. This indicates that high-affinity nitrate transporters in the roots of the halophyte are able to take up nitrate under high salinity; when potential competition with Cl^−^ ions is expected, the concentrations of Cl^−^ ions are nearly three orders of magnitude higher than concentrations of nitrate.

The salinity treatment from 0 to 750 mM NaCl influenced the concentrations of other measured ions in *S. altissima* plants. For high nitrate supply, chloride contents increased nearly linearly in all plant organs with increasing NaCl concentrations in the nutrient solution, while for low nitrate, the concentrations of chloride increased more slowly with signs of saturation at 750 mM NaCl (Figure 2c,d). Lower concentrations of chloride in *S. altissima* roots and higher in leaves under high nitrate supply, up to twofold at 750 mM NaCl, indicate that the halophyte accumulates chloride ions in leaf vacuoles, confirming the earlier results [33]. The deficit of nitrate limited growth and distorted the strategy. Chloride contents were similar for all the organs (apart at 500 mM NaCl) (Figure 2d). Sodium contents in *S. altissima* organs also increased nearly linearly as the concentration of sodium in the nutrient solution increased up to 750 mM, for both high and low nitrogen supply with two-and-more-fold lower Na^+^ in roots than in leaves (Figure 3a,b). It supported again the preferences of the halophyte to accumulation of ions in leaf vacuoles [33]. Concentrations of potassium and K^+^-to-Na^+^ ratios are more indicative for characterizing the development of salinity stress ([41,42]; proved for *Arabidopsis*–*Thellungiella* pair in [43]; for varieties of pepper in [44]; etc.). The loss of potassium from plant roots under salinity is assumed as one of determinants for salinity tolerance (e.g., reviewed in [42,45]). Potassium concentrations were higher under high nitrate without salinity in all the studied organs of *S. altissima* (Figure 3c,d). Rising salinity to 250 mM NaCl dropped the K^+^ concentrations under high nitrate in all organs, with a higher decrease in leaves and stems (Figure 3c). The further increase in salinity did not change the K^+^ concentrations, suggesting that the plants are adapted to the new high salinity environment. At low nitrate, the potassium concentration dropped in leaves only and remained about the same as under high nitrate under all the salinity treatments (0–750 mM) (Figure 3d). The K^+^ concentrations under salinity were highest in roots for high nitrate and in roots and stems under low nitrate, suggesting the active role of the organs in ion uptake and transport (Figure 3c,d).

In the present study, the coding sequences of two genes, *SaNRT2.1* and *SaNRT2.5,* for high-affinity nitrate transporters of the NRT2 family were cloned from euhalophyte *S. altissima*. Newly identified nitrate transporters from *S. altissima*, SaNRT2.1 and SaNRT2.5, are the putative orthologs of high-affinity nitrate transporters AtNRT2.1 and AtNRT2.5 from *A. thaliana*. The phylogenetic bioinformatic analysis demonstrated that the proteins SaNRT2.1 and SaNRT2.5 exhibit higher levels of homology to the corresponding proteins from the plants of family Amaranthaceae—*Amaranthus tricolor*, *Beta vulgaris*, *Chenopodium quinoa*, and *Spinacea oleracea* (Figure 5a). At the same time, the similarity of amino acid sequences between proteins SaNRT2.1 and SaNRT2.5 is not that high (54%). SaNRT2.1 demonstrated higher similarity in amino acid sequences to orthologs from *A. thaliana* (75%) than to SaNRT2.5 (Table 1). SaNRT2.5 in turn demonstrated a modest similarity to all corresponding orthologs from *A. thaliana*.

The topological models of SaNRT2.1 and SaNRT2.5 proteins (Figure 5b) predict 12 transmembrane domains in each of the proteins, corresponding to topological of proteins belonging to the MFS (major facilitator superfamily) with 12 transmembrane domains. Proteins of this superfamily are involved in the uptake of nitrate from soil by plant roots.

All NRT2s are high-affinity transporters. When the available nitrate is low, the high-affinity transport system is activated and plays a leading role in nitrate uptake by a plant [39,46]. The observed changes in expression of *SaNRT2.1* and *SaNRT2.5* under salinity and different (high and low) nitrate concentrations in the nutrient solution (Figure 8) point to potential and highly probable participation of the high-affinity transporters SaNRT2.1 and SaNRT2.5 in nitrate uptake by *S. altissima* plants under salinity. At the same time, the expression patterns of the genes for the two transporters significantly differ, which could be linked to the functional differences between SaNRT2.1 and SaNRT2.5 in intact *S. altissima* plants. When NaCl is absent in the medium, the gene *SaNRT2.1* is expressed at comparable levels in all studied organs of *S. altissima*; concentration of nitrate in the nutrient solution (low or high) did not essentially influence the level of expression (Figure 7a). On the other hand, the gene *SaNRT2.5* is expressed almost only in the roots; its level of expression is sharply increased under low nitrate (Figure 7b). An increase in NaCl in the nutrient solution from 0 to 750 mM under low nitrate increased the expression of both *SaNRT2.1* and *SaNRT2.5*, especially for roots, from 15 to 150 times at 500 mM NaCl, correspondingly. The expression level of *SaNRT2.5* exceeds the expression level of *SaNRT2.1* in roots by an order of magnitude (Figure 8a,b). Based on the analysis of the gene expression, it is highly likely that transporter SaNRT2.5 is a main player in ensuring effective nitrate uptake by roots and functions as an essential nitrate transporter to support the growth of adult halophyte plants under nitrogen deficiency. The two–threefold increase in expression of the nitrate transporter gene *SsNRT2.1* under salinity of 200 mM NaCl and 500 mM NaCl at the background of low (0.5 mM) nitrate was also demonstrated for euhalophyte *Suaeda salsa* [31]. The discovered regular patterns of *SaNRT2.1* and *SaNRT2.5* expression under NaCl conditions were similar to the patterns of expression for homologous genes in *A. thaliana*. In particular, four *Arabidopsis* genes, *AtNRT2.1*, *AtNRT2.4*, *AtNRT2.5* and *AtNRT2.6* showed strong preferential expression in the roots while conditions of low nitrate treatment significantly upregulated expression of *AtNRT2.1*, *AtNRT2.4* and *AtNRT2.5* [6,10,11,12]. Nevertheless, under low nitrate conditions, AtNRT2.1 is the main transporter for nitrate uptake and transport in roots [7,11]. The expression of *AtNRT2.5* is highly induced after a long period of starvation. Under these conditions, *AtNRT2.5* becomes the most abundantly expressed gene of the AtNRT2 family in roots and leaves, although *AtNRT2.5* transcripts were mainly present in roots and at much lower levels in shoots [6,11,47]. *AtNRT2.5* expression decreased under conditions of sufficient nitrate supply [7,12]. The same pattern was observed in our experiments for *SaNRT2.5* (and *SaNRT2.1*) expressed in roots (Figure 8a,b).

A direct attempt to prove the nitrate transporting function of SaNRT2.1 and SaNRT2.5 proteins from *S. altissima* was chosen using the heterologous expression system. The mutant strain *Δynt1* of yeast *H. polymorpha* is lacking the only nitrate transporter of the organism. Hence, the growth of the *Δynt1* mutant is suppressed at medium with nitrate as a single nitrogen source while the heterologously expressed nitrate transporters *AtNPF6.3* from *A. thaliana* and *SaNPF6.3* from *S. altissima* was effective in the growth rescue of the mutant [35,48]. Unfortunately, the heterologous expression of *SaNRT2.1* or *SaNRT2.5* genes in the yeast mutant did not rescue its growth at medium with nitrate. The same result was obtained when barley *HvNRT2.1* and *HvNRT2.2* cDNAs were used to complement a *Δynt1* mutant of *H. polymorpha* [39]. This complementation was only partial because, for unknown reasons, the nitrate uptake activity of the transformants was very low compared to the wild type [39]. The reasons for this absence of complementation could be multiple. The simplest ones are that the level of synthesized proteins SaNRT2.1 or SaNRT2.5 is too low in the heterologous systems, such that they are not delivered properly to the cellular yeast membrane, misfolded or not translated at all. A more elegant explanation comes from the fact that some proteins of NRT2 family require partner protein from the family NAR2/NRT3 for their proper functionality as nitrate transporters [47,49,50]. The interaction of NRT2 and NAR2 was demonstrated to enhance the nitrate transport activity of a high-affinity transport system [51,52]

There are, however, some NRT2 proteins that apparently function without these accessory proteins [50]. So, this unsuccessful complementation of yeast *Δynt1* mutant by heterologous expression of *SaNRT2.1* or *SaNRT2.5* genes could be explained by the omitted protein of family NAR2/NRT3 from *S. altissima* in the yeast heterologous expression system. So far, none of the genes for NAR2/NRT3 proteins have been cloned and studied in *S. altissima*. Further research is needed to elucidate the reasons for lack of activity of SaNRT2.1 and SaNRT2.5 in the yeast heterologous expression system. The identification and characterization of NAR proteins from *S. altissima* and understanding of their potential interactions with SaNRT2.1 and SaNRT2.5 is one of the ways in this direction.

## 4. Material and Methods

### 4.1. Plant Material

Seeds of *Suaeda altissima* (L.) Pall. were harvested from the plants growing on the shores of the salt lake Elton (Volgograd region, Russia). Seeds were germinated in wet sand at 24 °C. Fourteen days after germination, the seedlings were transferred to aerated Robinson–Downton nutrient solution (NS) [53] in 3 L vessels, 4 plants per vessel and 3 vessels per experimental point. NS was supplied with nitrate and chloride as KNO_3_ and NaCl, respectively. NS contained 0.5 mM NO_3_^−^ (low-nitrate medium) or 15 mM NO_3_^−^ (high-nitrate medium) and four increasing Cl^−^ concentrations (0, 250, 500, 750 mM) for each NO_3_^−^ condition. Plants were grown in hydroponic conditions at 24 °C, 60–70% relative humidity and under 16/8 h light/dark cycle for 4 weeks (young plants) or 6 weeks (adult plants), depending on the type of experiment. The plants were illuminated with high-pressure sodium lamps DNaZ_400 (Reflux, Novocherkassk, Russia) at a light flux of 300 µmol photons m^−2^ s^−1^. To study the long-term salinity effects on the growth characteristics of *Suaeda* plants and expression of the nitrate transporter genes in *Suaeda* organs, NaCl was added to the nutrient solution on the 7th day after the transfer of the seedlings from the wet sand to the vessels. To avoid salt shock, NaCl was added gradually in increments of 50 or 100 mM per day, up to the final concentrations of 250, 500 or 750 mM; no NaCl was added to the NS for control plants.

All chemicals used in this study for preparing Robinson–Downton nutrient solution [53] were of PTC (plant culture-tested) grade or molecular biology grade and manufactured by Central Drug House (P) Ltd. (New Delhi, India). Sodium chloride (extra-pure grade) was from Sisco Research Laboratories Pvt. Ltd. (Mumbai, India).

### 4.2. Yeast Strain and Vectors Used in This Study

Methylotrophic yeast *Hansenula polymorpha* double-auxotrophic strains DL-1 (*leu2 ura3* genotype) (wild-type strain, WT strain) and yeast integrative vectors pCCUR2 and pCHLX were used in this study. The strain DL-1 (*leu2 ura3*) was transformed with plasmids pCCUR2 and pCHLX carrying the *URA* and *LEU* genes, respectively, to ensure the growth of the yeast strains without additional nitrogen sources, leucine and uracil, when performing complementation tests. Plasmids pCCUR2 and pCHLX were kindly provided by Michael Agafonov (Federal Research Center “Fundamentals of Biotechnology”, Russian Academy of Sciences, Moscow, Russia). Yeast cells were transformed by the lithium method [54] or by electroporation [55] using an Eppendorf device (Eppendorf, Framingham, MA, USA). *H. polymorpha* mutant strain *Δynt1* with a deleted *YNT1* gene encoding the only high-affinity nitrate transporter in *H. polymorpha* was produced from the wild-type *H. polymorpha* strain DL-1 (*leu2 ura3*) by us earlier [48]. The mutant strain *Δynt1* (*ynt1: BleoR/ZeoR, leu2, ura3*) was also transformed with pCCUR2 and pCHLX integrative plasmids carrying the *URA* and *LEU* genes, respectively, to ensure the growth of the yeast strains without additional nitrogen sources in the selective media, namely, leucine and uracil, when performing complementation tests.

### 4.3. Plant Organ Fresh and Dry Weight Analysis

Quantitative analyses of the overall growth parameters of *S. altissima* plants were made after 6 weeks of growing plants in hydroponics. Root, stem and leaf fresh weights (FWs) were measured after removal of the entire plant from the hydroponics system. The respective dry weights (DWs) were measured after drying plant material in drying oven Binder FD115 (Tuttlingen, Germany) at 90 °C for 48 h.

### 4.4. Determination of NO_3_^−^ and Cl^−^ Contents in S. altissima Organs

Water extracts from the samples of dried *S. altissima* organs (roots, stems and leaves) were prepared by incubating 100 mg of homogenized samples in 10 mL aliquots of boiling deionized water for 10 min. Concentrations of Cl^−^ and NO_3_^−^ in the extracts were determined using ion-selective electrodes (respectively, Elite-261 and Elite-021, Niko-Analit, Moscow, Russia).

### 4.5. Extraction of Total RNA from Plant Material and First-Strand cDNA Synthesis

For total RNA extraction *S. altissima* plants after 4 weeks of growing in hydroponics were used. Plant organs (roots, leaves, stems) were sampled (approximately 1 g fresh weight of each sample), frozen in liquid nitrogen and stored at −80 °C for the further use.

Total RNA from *S. altissima* plant organs was isolated by the hot phenolic method [56] and used as a template for the total first-strand cDNA synthesis. For amplification of the 3′- and 5′-ends of the *SaNRT2.1* and *SaNRT2.5* transcripts by the Step-Out RACE method, the first strand of cDNA was synthesized on the total RNA template isolated from *Suaeda* roots using MINT revertase (Evrogen, Moscow, Russia). Full-length cDNAs of the *SaNRT2.1* and *SaNRT2.5* genes were also amplified on the total RNA template isolated from *Suaeda* roots. To obtain full-length cDNAs of the *SaNRT2.1* and *SaNRT2.5* and quantify the representation of the gene transcripts in *S. altissima* organs, first-strand cDNA synthesis was performed on the total RNA templates using (dT)15 primer and MMLV revertase (Evrogen, Moscow, Russia).

### 4.6. Primer Design

Primer for qPCR-RT experiments were designed using primer Blast software, version 4.1.0 (https://www.ncbi.nlm.nih.gov/tools/primer-blast/, accessed on 22 March 2024). Other primers used were selected using SnapGene Viewer software 5.0.8 (https://www.snapgene.com/snapgene-viewer, accessed on 29 July 2023). All primers used are listed in Table S1.

### 4.7. Identification of the Full-Length SaNRT2.1 and SaNRT2.5 Coding Sequences

Partial coding sequences (the middle fragments) of the *SaNRT2.1* and *SaNRT2.5* genes were obtained by us previously (GenBank ID: MK580128.1 and MK580129.1, accordingly) [37]. Here, based on these sequences, the forward and reverse primer sets were designed for amplification of the 5′- and 3′-end sequences of *SaNRT2.1* and *SaNRT2.5* cDNAs. The 5′- and 3′-end sequences of the targeted cDNAs were obtained using Step-Out RACE technology (kit #SKS03, Evrogen, Moscow, Russia), following the manufacturer’s protocol. The cDNA fragments were amplified on the total cDNA template using Encyclo DNA polymerase (#PK002, Evrogen, Moscow, Russia). The 5′-end sequence of *SaNRT2.1* cDNA was amplified with primers SaNRT2.1_R (round 1) and SaNRT2.1_R1 (round 2). The 3′-end sequence of SaNRT2.1 cDNA was amplified with SaNRT2.1_F (round 1) and SaNRT2.1_F1 (round 2). Similarly, 5′-end and 3′-end fragments of *SaNRT2.5* cDNA were amplified with primers SaNRT2.5_R and SaNRT2.5_F (round 1), SaNRT2.5_R2 and SaNRT2.5_F2 (round 2). The primers Mix1 (round 1) and Mix2 (round 2) from the manufacturer kit were also used for amplification of *SaNRT2* fragments.

The amplicons obtained were cloned into pAL2-T vector (Evrogen, Moscow, Russia) for replication in *E. coli* cells and the following sequencing. Subsequently, overlapping 5′- and 3′-ends of *SaNRT2.1*, 5′- and 3′-ends of *SaNRT2.5* cDNAs were in silico assembled using SnapGene software 5.0.8 (https://www.snapgene.com/snapgene-viewer, accessed on 29 July 2023). Two resulting sequences were obtained containing open reading frames of 1575 bp (*SaNRT2.1*) and 1503 bp (*SaNRT2.5*), respectively. These sequences were used for the design of primers for the amplification of full-length *SaNRT2.1* and *SaNRT2.5* cDNAs on the total first-strand cDNA template. The full-length *SaNRT2.1* and *SaNRT2.5* coding sequences were amplified from the total first-strand cDNA using primer pairs SaNRT2.1b_F1 and SaNRT2.1b_R, and SaNRT2.5a_F and SaNRT2.5a_R1, respectively. All amplicons obtained were analyzed by electrophoresis in 1% agarose gel.

The full-length *SaNRT2.1* and *SaNRT2.5* cDNAs were cloned into vector pCHLX [57] under the control of the inducible nitrate reductase (NR) promoter *pYNR1* and terminator *tYNR1* of *H. polymorpha*. Promoter *pYNR1* and terminator *tYNR1* sequences were amplified from the *H. polymorpha* genomic DNA template using primer pairs pYNR1_F and pYNR1_R, and tYNR1_F and tYNR1_R. The first 10 cycles of amplification of the promoter, terminator and gene coding sequences were performed using Encyclo polymerase (No. PK002, Evrogen, Moscow, Russia); the next 25 cycles were performed using CloneAmp HiFi PCR Premix kit (No. 639298, Clontech, Mountain View, CA, USA). The pCHLX vector was linearized in the Hind III and EcoRI restriction sites and ligated with the synthesized *pYNR1*, *tYNR1* and *SaNRT2.1*/*SaNRT2.5* sequences using a Gibson assembly kit (No. E5510, SkyGen, NEB, Ipswich, MA, USA) to produce the pCHLX-pYNR1-SaNRT2.1-tYNR1 or pCHLX-pYNR1-SaNRT2.5-tYNR1 constructs (further denoted as pCHLXSaNRT2.1 or pCHLXSaNRT2.5, respectively). The cloned sequences, *SaNRT2.1* (1575 bp) and *SaNRT2.5* (1503 bp), were verified by sequencing and deposited in GenBank (*SaNRT2.1* ID: OR909030.1; *SaNRT2.5* ID: OR828748.1).

### 4.8. Quantitative Analysis of SaNRT2.1 and SaNRT2.5 Transcripts in S. altissima Organs

Quantitative analysis of *SaNRT2.1* and *SaNRT2.5* transcripts was performed by qRT-PCR using a LightCycler^®^ 96 System (Roche Diagnostics Corporation, Indianapolis, IN, USA). The cDNA templates for the amplification of *SaNRT2.1* and *SaNRT2.5* fragments were synthesized on the total RNAs templates, isolated from the organs of *S. altissima* plants grown in the NS supplied with various nitrate and NaCl concentrations. A ready-to-use reaction mixture with intercalating dye SYBR Green I for real-time PCR (Evrogen, Moscow, Russia) was used. The *S. altissima* gene of elongation factor 1 alpha Sa*eEF1alpha* (GenBank ID: MN076325.1) was used as an internal control. The expression of this housekeeping gene has been shown to be constitutive within the different plant organs under changing experimental conditions [58]. To amplify the Sa*eEF1alpha* fragment, SaeEF1alfa_F1 and SaeEF1alfa_R1 primers were used. The results obtained were processed using LightCycler 96SW 1.1 software. The relative expression profile was calculated by the 2^−∆∆CT^ method. For the amplification of SaNRT2.1 and SaNRT2.5 fragments, the primer pairs SaNRT2.1_F1 and SaNRT2.1_R1, and SaNRT2.5_F2 and SaNRT2.5_R2 were used. The results are based on three biological and three analytical replicates.

### 4.9. Cultivation of H. polymorpha WT Strain and Δynt1 Transformants

Cells of *H. polymorpha* WT strain and mutant *Δynt1* strain were grown in a rich YPD medium (1% yeast extract, 2% peptone, 2% glucose). After co-transformation with pCHLX and pCCUR2 vectors, yeast cells were grown on a minimal synthetic SD medium (0.17% yeast nitrogen base without amino acids and ammonium sulfate, 2% glucose) with the addition of 0.5% (NH_4_)_2_SO_4_ as a nitrogen source. All solid media contained 2% agar.

For complementation assay, at first, *H. polymorpha* WT and *Δynt1* strains co-transformed with pCCUR2 and pCHLX vectors (with or without SaNRT2.1/SaNRT2.5 insert) were grown in 10 mL of minimal SD medium containing 0.5% ammonium sulfate overnight at 37 °C. Then, the obtained cultures were centrifuged for 5 min at 2500 *g*, the precipitates were washed with sterile water, resuspended in the water and 2 μL suspension samples were plated on agarized SD medium containing KNO_3_ instead of ammonium sulfate at concentrations ranging from 0.2 to 5 mM. The resulting samples were incubated at 37 °C for 2–3 days until colonies appeared.

All manipulations with *H. polymorpha* were performed according to the protocols generally accepted for the yeast [55]. Yeast transformants were selected on minimal selective media in the absence of leucine and/or uracil. Transformants that contained the insertion in the genome were validated by PCR with Hp_DL-1_Chr1_R primers for genomic DNA and standard M13_F primers for pCHLX vectors.

### 4.10. Bioinformatic Analysis

The online translation of nucleotide *SaNRT2.1* and *SaNRT2.5* encoding sequences to the amino acid ones was carried out using online service at ExPASy portal (http://web.expasy.org/translate/, accessed on 22 March 2024). Molecular weights of proteins SaNRT2.1 and SaNRT2.5 were calculated at the same portal (http://web.expasy.org/protparam/, accessed on 22 March 2024). The classification of the identified transporters within NRT2 family was determined using InterPro v.98.0 (http://www.ebi.ac.uk/interpro/, accessed on 22 March 2024) and also with the aid of the algorithm Protein BLAST (Basic Local Alignment Search Tool) at the NCBI portal (https://blast.ncbi.nlm.nih.gov/Blast.cgi, accessed on 22 March 2024). Cellular localization of SaNRT2.1 and SaNRT2.5 was predicted using online service WoLF PSORT II at GenScript (https://www.genscript.com/wolf-psort.html, accessed on 22 March 2024).

Multiple sequence alignment of amino acid sequences of NRT2 proteins was performed using Clustal Omega software (https://www.ebi.ac.uk/jdispatcher/msa/clustalo, accessed on 25 March 2024) and visualized with Jalview software, version 2.11.2.7 (https://www.jalview.org/, accessed on 25 March 2024). A phylogenetic analysis of NRT2 family proteins was carried out using Molecular Evolutionary Genetic Analysis (MEGA) 11 software (version 11, https://www.megasoftware.net/, accessed on 14 March 2024), using the maximum likelihood method based on the Jones–Taylor–Thornton model [59] (1000 bootstrap replications were performed). NRT2 protein sequences for comparative analysis were extracted from the NCBI portal (https://www.ncbi.nlm.nih.gov/protein, accessed on 14 March 2024).

Protein topologies were predicted by DeepTMHMM software (version 1.0.24, https://dtu.biolib.com/DeepTMHMM, accessed on 12 March 2024) [60].

### 4.11. Statistical Analysis

The results presented in Figure 1, Figure 2, Figure 3, Figure 7 and Figure 8 were deduced from three biological replicates and each of them was performed in three analytical replicates. The significant difference was analyzed by using Student’s *t*-test. A *p*-value < 0.05 was considered to be statistically significant. Standard deviations are given in the figures.

## 5. Conclusions

Concluding, two genes of high-affinity nitrate transporters, *SaNRT2.1* and *SaNRT2.5*, were cloned from the euhalophyte *Suaeda altissima*, which is able to grow and proliferate at 1 M NaCl. The expression patterns of *SaNRT2.1* and *SaNRT2.5* were studied for the plants grown under low (0.5 mM) or high (15 mM) nitrate with salinity ranging from 0 to 750 mM NaCl. *SaNRT2.1* was expressed in all organs when *SaNRT2.5* was expressed exclusively in roots. Under low nitrate in medium, salinity increased the expression of both genes: at 500 mM NaCl, *SaNRT2.1* peaked in the roots with a 15-fold rise; *SaNRT2.5* peaked in the roots, rising 150-fold. An attempt to demonstrate nitrate transporting activity of SaNRT2.1 or SaNRT2.5 in the yeast heterologous expression system was not successful; hence, future research is aimed at finding the partner protein of the NAR2/NRT3 family from *S. altissima* and functional characterization of the nitrate transporters SaNRT2.1 and SaNRT2.5.

## Figures and Tables

**Table 1:** Identities of aa sequences of SaNRT2.1, SaNRT2.5 and NRT2 proteins of *A. thaliana* calculated in BLAST, NCBI (https://blast.ncbi.nlm.nih.gov/Blast.cgi, accessed on 25 March 2024). Identities are expressed as a percentage.

	SaNRT2.1	AtNRT2.1	AtNRT2.2	AtNRT2.3	AtNRT2.4	AtNRT2.5	AtNRT2.6	AtNRT2.7
**SaNRT2.5**	53.37	53.46	53.27	54.38	54.21	65.36	55.47	49.79
**AtNRT2.1**	74.48		87.26	69.04	84.23	59.75	68.28	46.52
**AtNRT2.2**	71.76	87.26		66.8	80.98	58.58	66.93	46.53
**AtNRT2.3**	68.68	69.04	66.8		71.76	57.02	89.3	47.98
**AtNRT2.4**	73.9	83.24	80.98	71.76		58.14	71.16	47.17
**AtNRT2.5**	58.21	59.75	58.58	57.02	58.14		57.76	49.9
**AtNRT2.6**	69.19	68.28	66.93	89.3	71.16	57.76		47.75
**AtNRT2.7**	46.86	46.52	46.53	47.98	47.17	49.9	47.75	

## Data Availability

All data included in this study are available upon request by contact with the corresponding authors. The seeds of *Suaeda altissima* are available from the authors on request. The cloned *SaNRT2.1* and *SaNRT2.5* coding sequences were deposited in GenBank (*SaNRT2.1* ID: OR909030.1; *SaNRT2.5* ID: OR828748.1).

## Supplementary Materials

The supporting information can be downloaded at: https://www.mdpi.com/article/10.3390/ijms25115648/s1.
