# Supplementary material for: Novel Proteins of the High-Affinity Nitrate Transporter Family NRT2, SaNRT2.1 and SaNRT2.5, from the Euhalophyte Suaeda altissima: Molecular Cloning and Expression Analysis
Source: Int J Mol Sci. 2024 May 22;25(11):5648. doi: 10.3390/ijms25115648 (PMC11171637; doi:10.3390/ijms25115648)
Supplement: Supplementary file 1 [file ijms-25-05648-s001.zip › ijms-2965248-supplementary.pdf]

Supplementary materials

Table S1. List of the primers used in the research.

Primer name	Sequence (5'-3')	Gene
qRT-PCR		
SaNRT2.1_F1	GTGCTTCTGTATGGATACTCT	SaNRT2.1
SaNRT2.1_R1	ATTCCAGCCGTGTGTAG	
SaNRT2.5_F2	TACTGGATGAGCTCCATGTT	SaNRT2.5
SaNRT2.5_R2	ACATTGTAGATTAGAGGCATCAC	
SaeEF1alfa_F1	TGAGATGTGTGGCAATCC	SaeEF1α
SaeEF1alfa_R1	GTTGCTTCTGACTCCAAGAAT	
5'-RACE (round 1)		
SaNRT2.1_R	GTAGCAGAAGCCACCCTACGC	SaNRT2.1
SaNRT2.5_R	AATCCACCAGGTCCATATTCTTCC	SaNRT2.5
3'-RACE (round 1)		
SaNRT2.1_F	CCTACGGTCTTTTGCATGTCGTT	SaNRT2.1
SaNRT2.5_F	ATGAGGGCATTTCATCTATGTTGG	SaNRT2.5
5'-RACE (round 2)		
SaNRT2.1_R1	ATTCCAGCCGTGTGTAG	SaNRT2.1
SaNRT2.5_R2	ACATTGTAGATTAGAGGCATCAC	SaNRT2.5
3'-RACE (round 2)		
SaNRT2.1_F1	GTGCTTCTGTATGGATACTCT	SaNRT2.1
SaNRT2.5_F2	TACTGGATGAGCTCCATGTT	SaNRT2.5
Amplification of full-length cDNA		
SaNRT2.1b_F1	ATGGCTGGTGAGCCGG	SaNRT2.1
SaNRT2.1b_R	CTATATTTGGTTAGAGTTGGGGCTTTGTG	
SaNRT2.5a_F	ATGGCATCAACAAATGAAGAATTTGC	SaNRT2.5
SaNRT2.5a_R1	TTAGACACGATTAACTGTACTGCCT	
Amplification of pYNR1		
pYNR1_F	GGTCGACGGTATCGATAAGCTTATGGGGCTC CATATATCGTATGAC	SaNRT2.1
pYNR1-SaNRT2.1b-R1	CGGCTCACCAGCCATGAATTCTACAACTATC CAAAGTTCGTGAGG	
pYNR1_F	GGTCGACGGTATCGATAAGCTTATGGGGCTC CATATATCGTATGAC	SaNRT2.5
pYNR1-SaNRT2.5a_R	CTTCATTTGTTGATGCCATGAATTCTACAACT ATCCAAAGTTCGTGAGG	
Amplification of tYNR1		
tYNR1-SaNRT2.1b_F	CCCCAACTCTAACCAAATATAGCTTAAGCCT AAGCGAAATCGAAATCAAAACT	SaNRT2.1
tYNR1_R	CCCGGGCTGCAGGAATTAATTCAGTATTTCA GAATCATGACCCC	
tYNR1_SaNRT2.5a_F1	AGTACAGTTAATCGTGTCTAAGCTAGCCCTA AGCGAAATCGAAATCAAAACT	SaNRT2.5
tYNR1_R	CCCGGGCTGCAGGAATTAATTCAGTATTTCA GAATCATGACCCC	
Screening of H. polymorpha transformants		
Hp_DL-1_Chr1_R	TTGGTACCACCGCACTTCTC	
M13_F	GTTGTAAAACGACGGCCAGTG	

Table S2. List of proteins from the phylogenetic tree of Figure 5a.

Plant species	Protein name	GenBank ID
Arabidopsis thaliana	AtNRT2.1	NP_172288.1
	AtNRT2.2	NP_172289.1
	AtNRT2.3	NP_200886.1
	AtNRT2.4	NP_200885.1
	AtNRT2.5	NP_172754.1
	AtNRT2.6	NP_190092.1
	AtNRT2.7	NP_196961.1
Amaranthus tricolor	AtrNRT2.1	XP_057535583.1
	AtrNRT2.5	XP_057530035.1
Beta vulgaris	BvNRT2.1	XP_010685329.1
	BvNRT2.4	XP_048504348.1
	BvNRT2.5	XP_010695818.2
Brassica napus	BnNRT2.6	XP_013677304.1
	BnNRT2.7	XP_013726185.2
Brassica rapa	BrNRT2.6	XP_009107824.2
	BrNRT2.7	XP_009125986.2
Cannabis sativa	CsaNRT2.5	XP_030496215.2
Capsella rubella	CrNRT2.5	XP_006307313.1
	CrNRT2.6	XP_006293136.1
	CrNRT2.7	XP_006286495.1
Cucumis sativus	CsNRT2.1	AZP53714.1
	CsNRT2.2	AGO64298.1
	CsNRT2.3	AGO64299.1
	CsNRT2.4	NP_001274401.1
	CsNRT2.5	NP_001295862.1
Chenopodium quinoa	CqNRT2.1	XP_021716511.1
	CqNRT2.5	XP_021713512.1
Eutrema salsugineum	EsNRT2.5	XP_006417172.1
	EsNRT2.6	XP_006419073.1
Glycine max	GmNRT2.4	XP_003539195.1
Hordeum vulgare	HvNRT2.1	AAC49531.1
	HvNRT2.2	AAC49532.1
	HvNRT2.4	KAE8777997.1
	HvNRT2.5	KAE8819762.1
Medicago truncatula	MtNRT2.1	XP_013455974.2
	MtNRT2.7	KEH30005.1
Nicotiana sylvestris	NsNRT2.7	XP_009757883.1
Raphanus sativus	RsNRT2.5	XP_018449553.1
Solanum lycopersicum	SlNRT2.3	NP_001234127.1
	SlNRT2.7	XP_004233327.2
Spinacia oleracea	SoNRT2.1	XP_021865042.1
	SoNRT2.5	XP_021845686.1
Suaeda altissima	SaNRT2.1	WPS65192.1
	SaNRT2.5	WPH61290.1
Vigna angularis	VaNRT2.1	XP_017409445.1
